# Phenotypic Variation in Patients with Chronic Obstructive Pulmonary Disease in Primary Care

**DOI:** 10.1155/2016/8108717

**Published:** 2016-04-11

**Authors:** Emmylou Beekman, Ilse Mesters, Mark G. Spigt, Eva A. M. van Eerd, Rik Gosselink, Rob A. de Bie, Onno C. P. van Schayck

**Affiliations:** ^1^Department of Epidemiology, CAPHRI School for Public Health and Primary Care, Maastricht University Medical Centre, P.O. Box 616, 6200 MD Maastricht, Netherlands; ^2^Research Centre for Autonomy and Participation of Persons with a Chronic Illness, Zuyd Hogeschool, P.O. Box 550, 6400 AN Heerlen, Netherlands; ^3^Physical Therapy Section in Multidisciplinary Centre, ParaMedisch Centrum Zuid, Veestraat 28, 6134 VJ Sittard, Netherlands; ^4^Department of Family Medicine, CAPHRI School for Public Health and Primary Care, Maastricht University, P.O. Box 616, 6200 MD Maastricht, Netherlands; ^5^Department of Rehabilitation Sciences, Katholieke Universiteit Leuven, P.O. Box 1500, 3001 Heverlee, Belgium; ^6^Department of Respiratory Rehabilitation and Respiratory Division, University Hospital Leuven, P.O. Box 706, 3000 Leuven, Belgium

## Abstract

*Introduction*. Despite the high number of inactive patients with COPD, not all inactive patients are referred to physical therapy, unlike recommendations of general practitioner (GP) guidelines. It is likely that GPs take other factors into account, determining a subpopulation that is treated by a physical therapist (PT). The aim of this study is to explore the phenotypic differences between inactive patients treated in GP practice and inactive patients treated in GP practice combined with PT. Additionally this study provides an overview of the phenotype of patients with COPD in PT practice.* Methods*. In a cross-sectional study, COPD patient characteristics were extracted from questionnaires. Differences regarding perceived health status, degree of airway obstruction, exacerbation frequency, and comorbidity were studied in a subgroup of 290 inactive patients and in all 438 patients.* Results*. Patients treated in GP practice combined with PT reported higher degree of airway obstruction, more exacerbations, more vascular comorbidity, and lower health status compared to patients who were not referred to and treated by a PT.* Conclusion*. Unequal patient phenotypes in different primary care settings have important clinical implications. It can be carefully concluded that other factors, besides the level of inactivity, play a role in referral to PT.

## 1. Introduction

Physical activity is beneficial for patients suffering from chronic obstructive pulmonary disease (COPD). Physical activity can improve symptoms, quality of life, and physical and emotional participation in everyday activities [1], whereas a decline from moderate/high physical activity to low physical activity is associated with an increased mortality risk [2]. To describe the number of active or inactive COPD patients the physical activity norm for healthy persons is applied as the norm for physical activity in patients with COPD. To be considered sufficiently physically active, a healthy adult has to carry out moderate intense physical activities for at least five days a week, 30 minutes a day (Dutch standard) [3] or 20 min of vigorous-intensity physical activity on at least 3 days every week, or an equivalent combination, which can also be accumulated in shorter bouts of 10 min exercise (international standard) [3–7]. The extent to which this framework, on which to base recommendations for physical activity promotion, applies to patients with COPD is currently unknown [6]. Since no definite directives are available about how much physical activity COPD patients should carry out, the standard for healthy persons is used in health care [7].

Epidemiological data show that 84% of patients with COPD do not reach the Dutch standard for daily physical activity [8]. Systematic literature reviews conclude that the number of inactive patients with COPD is some 30% (range 17–43%) higher compared to inactive healthy adults [5, 9–11] and higher compared to other patients with chronic diseases like diabetes mellitus and rheumatoid arthritis [12]. Lower levels of physical activity were already present in the earlier stages of the disease, and an increasing severity of COPD was associated with a further decrease in physical activity [13].

The Dutch general practitioners (GPs) practice guidelines advice to refer patients with COPD to a physical therapist (PT) if they do not or cannot comply with the Dutch standard for physical activity due to dyspnoea or fear of dyspnoea [14]. Although inactivity is a referral criterion for physiotherapy, there is a discrepancy between the numbers of patients with COPD who are inactive (the earlier mentioned 84%) and those with COPD treated by a PT (27%) [15]. Indeed, patients may decide (not) to opt for physical therapy; for example, patients with COPD perceive their health condition (dyspnoea) as less severe compared to the objective degree of severity [16]. However, it is more likely that GPs take severity of obstruction, symptoms (dyspnoea), exacerbation risk, and presence of comorbidities into account, besides inactivity, to refer patients with COPD to physical therapy [1, 17]. Other patients' symptoms and perceived level of limitations may additionally play a role in referring patients to the PT [17]. Patients' perceptions of limitations are a stronger predictor of behaviour (like physical activity) than objective measures of limitation severity [18], as they contribute to the larger patients' burden of disease. From a patient perspective, COPD can be held responsible for disability that restricts many everyday activities, such as walking upstairs [19]. Hence, when assessing the patients' burden of COPD, patient reported outcome measures should be incorporated, for instance, the Clinical COPD Questionnaire (CCQ) that assesses a broader range of health status than dyspnoea only [20–22]. We hypothesise that the referral of patients to PT is based on the patients' burden of disease and that this is not necessarily coherent with the level of inactivity.

General practitioner's considerations to refer to physical therapy are likely to determine patient flow in primary care. GPs treat a wide spectrum of patients from less severe to very severe COPD. PTs however seem to treat a subpopulation of this spectrum. Although the descriptions of COPD populations in the literature are limited to in-patients or out-patients who are under supervision of pulmonary clinics [23, 24], PTs in primary care settings believe that they are involved with patients with a high burden of disease. Since physical therapists are expected to tailor their clinical reasoning and their choice for exercise therapy to the population that visits the PT [25], insight in the overall phenotype of their patients is crucial for PTs. Depending on the level of inactivity but also depending on other patient characteristics like the presence of comorbidities [25] and future risk of exacerbations [1], PTs may have to take into account extensive interdisciplinary consultation, adapted training intensity, or longer treatment duration. We hypothesise that patient phenotypes are unequal in different primary care settings.

This study explores the phenotypic differences between inactive patients treated in GP practice and inactive patients treated in GP practice combined with PT, with regard to patients' perceived health status, degree of airway obstruction, exacerbation frequency, and comorbidity. Additionally it provides an overview of the overall phenotype of patients with COPD in PT practice.

## 2. Methods

### 2.1. Participants

In 2012, cross-sectional data were collected in collaboration with ten multidisciplinary primary health care centres (collaboration “SGE”) providing care to 64,602 people in Eindhoven, Netherlands [26]. In this population, 1,248 patients were diagnosed with COPD, as registered with code R95 in accordance with the International Classification of Primary Care (ICPC) in the general practice patient documentation system. In December 2012, questionnaires were sent by post to all 1,248 patients.

### 2.2. Measurements

The questionnaire was developed by Maastricht University in collaboration with the participating health centres. It contained items regarding personal characteristics and disease severity (self-reported). Disease-related health status was measured with the Clinical COPD Questionnaire [27], addressing symptoms, functional state, and mental state (CCQ, rating from 0 “good” to 6 “bad”). General health status was measured with the first question on The Short Form Health Survey (SF36, rating from 1 “excellent” to 5 “poor”) [28]. Information regarding physical activity (Physical Activity questionnaire, rating from 0 “not physically active” to 8 “very physically active”) [29], smoking, and comorbidities (for 15 different disease categories) was collected. Exacerbation history was measured by an event-based approach (the number of hospitalisations and medication intake (0, 1, 2, 3, or 3>)). Whether patients were treated by a PT for COPD or another health condition was collected as well. Inactivity was defined as moderate intense physically active for less than five days a week (30 minutes a day) and vigorous-intense physically active for less than three days a week (20 minutes a day) or an equivalent combination. This corresponds with a score between zero and three on the Physical Activity questionnaire [7, 29] and is in agreement with the international standard for physical activity [3–6].

### 2.3. Data Analyses

From the questionnaires returned, individual anonymised data were used. Phenotypic variations in inactive patients with COPD and in all patients with COPD treated by a GP versus a PT were analysed, based on the patient reported outcome measures. The following factors were treated as categorical data: sex, Global Obstructive Lung Disease (GOLD stages I–IV) [1], general health status (SF36), comorbidity (yes or no for 15 different disease categories), exacerbation frequency (number of hospitalisations and medication intake (0, 1, 2, 3, or 3>)), and physical therapy treatment (yes or no). Age, disease-related health status (CCQ), physical activity (Physical Activity questionnaire), and smoking history (pack years) were treated as continues data. Double answers were treated as was specified in the original questionnaires. If not specified, the less favourable answer was taken (e.g., “GOLD 3” AND “GOLD 4” were replaced by GOLD 4; “1-2 days a week physical active” AND “3-4 days a week physical active” were replaced by 1-2 days a week; “25 cigarettes a day” AND “15 cigarettes a day” were replaced by 25 cigarettes).

Between-group differences (GP treatment versus GP combined with PT treatment) were analysed for the inactive population and for the whole population by crosstabs with Pearson *χ* ^2^ and odds ratios (OR) for sex, GOLD stage, presence of comorbidity, exacerbation frequency, and general health perception, with an independent *t*-test for age, and with the Mann-Whitney test for smoking, physical activity, and disease-related health status.

## 3. Results

**Figure 1:**
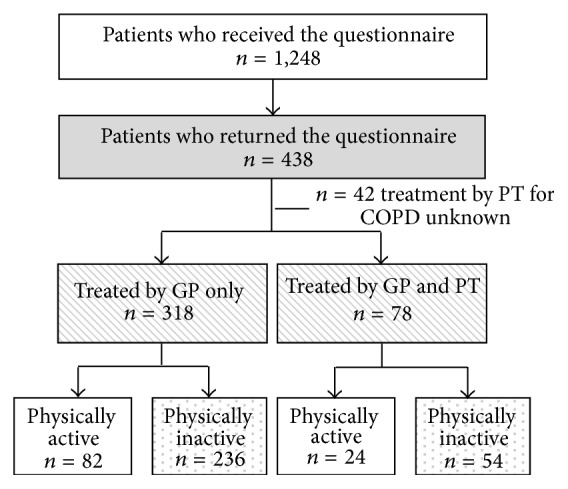
Flowchart of patients registered and treated in primary care. GP: general practitioner; PT: physical therapist; COPD: chronic obstructive pulmonary disease. Subgroups in the dotted boxes were compared for the first aim of this study. Subgroups in the striped boxes were compared for the second aim of this study.

Four hundred and thirty-eight completed questionnaires were returned, with a response rate of 35%. Missing data was treated by case wise deletion for each statistical run (41 in physical activity; 24 in smoking history; 4 in comorbidity; 126 in GOLD stage; 40 in exacerbations; 121 in health status; 42 in health perception; and 42 in treatment GP versus PT). Data was sampled independently from the populations being compared, with equal variances. According to the respondents, eighteen percent of the respondents were treated for COPD by a PT. Of those patients, 69% were physically inactive. In the group that did not receive PT, 74% were physically inactive. Moreover, a total of 73% of patients with COPD registered by the GP were physically inactive.  Figure 1 presents the flow of patients and the subgroups analysed.

### 3.1. Phenotype of Inactive Patients in Primary Care

Table 1 presents characteristics of all inactive patients, based on patient reported outcome measures. Patients who were referred to PT did not differ significantly from patients who were treated by a GP only regarding the demographic characteristics sex (*χ* ^2^(1) = 0.53, *p* = 0.55) and age (*t*(287) = −0.36, *p* = 0.72).

Inactive patients treated by GP combined with PT reported a statistically significant higher degree of airway obstruction compared to patients treated by a GP only (*χ* ^2^(3) = 49.10, *p* < 0.0001). Patients with GOLD II or higher were 9 times more likely to be treated by a PT compared to patients with GOLD I; for patients with GOLD III or higher it was 10 times more likely.

Also, higher comorbidity rates were reported in the group treated by GP combined with PT, but only vascular disease was significantly more present (*χ* ^2^(1) = 5.77, *p* = 0.020); those with an additional vascular disease were 2.3 times more likely to be treated by a PT. Other disease groups, like neoplasms, musculoskeletal, skin, endocrine, digestive, or neurological disease, were not statistically different between the groups. Moreover, these subgroups were very small and therefore not shown in Table 1.

Significantly higher exacerbation rates were reported by patients treated by PT (*χ* ^2^(3) = 17.02, *p* = 0.001). The chance for treatment by a PT increased gradually with higher exacerbation frequencies (OR = 2.8 with one or more exacerbations; OR = 3.2 with two or more exacerbations; OR = 3.3 with three or more exacerbations).

General health perception was significantly lower in the group treated by PT (*χ* ^2^(2) = 16.44, *p* < 0.0001); those who rate their general health as poor or moderate were almost two times more likely to be treated by GP and PT combined. Comparably, disease-related health status was significantly lower based on the total CCQ scale (*U* = 5762.500, *p* < 0.0001) and based on the subscales for symptoms (*U* = 6541.500, *p* = 0.004), functional state (*U* = 7904.000, *p* < 0.0001), and mental state (*U* = 5016.500, *p* = 0.038).

### 3.2. Phenotype of Patients in PT Practice

Characteristics of patients treated by PT versus patients treated by a GP only can be found in Table 2. All patients (active and inactive) who received PT did not differ significantly from patients who did not receive PT regarding the demographic characteristics sex (*χ* ^2^(1) = 0.71, *p* = 0.45) and age (*t*(393) = −1.06, *p* = 0.29).

Patients treated by both a GP and a PT reported a statistically significant higher degree of airway obstruction compared to patients treated by a GP only (*χ* ^2^(3) = 79.75, *p* < 0.0001). Patients with GOLD II or higher were 15 times more likely to be treated by a PT compared to patients with GOLD I.

Also, high comorbidity rates were reported in the group treated by PT (Table 2), but only vascular disease was significantly more present (*χ* ^2^(1) = 7.51, *p* = 0.009); those with an additional vascular disease were 2.7 times more likely to be treated by a PT.

Significantly higher exacerbation rates were shown in patients treated by PT (*χ* ^2^(4) = 35.91, *p* < 0.0001). The chance for treatment by a PT increased gradually with higher exacerbation frequencies (OR = 3.4 with one or more exacerbations; OR = 3.7 with two or more exacerbations; OR = 3.8 with three or more exacerbations).

General health perception was significantly lower in the group treated by PT (*χ* ^2^(2) = 23.71, *p* < 0.0001); those who rate their general health as poor or moderate were four times more likely to be treated by GP and PT combined. Also, disease-related health status was significantly lower based on the total CCQ scale (*U* = 11923.000, *p* < 0.0001) and based on the subscales for symptoms (*U* = 13806.500, *p* < 0.0001), functional state (*U* = 16485.000, *p* < 0.0001), and mental state (*U* = 9962.000, *p* = 0.004).

Patients treated by a PT were significantly more physically active in their daily life (*U* = 9438.000, *p* = 0.001) but had a significantly higher history of pack years (*U* = 8863.500, *p* = 0.004).

## 4. Discussion

This study showed that there are phenotypic differences between patients with COPD in primary care. More specifically, inactive patients treated in GP practice combined with PT had a higher degree of airway obstruction, more exacerbations, and more vascular comorbidity and a lower health status was reported. It may be that patients who are referred to PT have a higher burden of disease compared to patients who are not referred to a PT. Moreover, this study gave an overview of the overall phenotype of patients with COPD in PT practice. We showed that the group of patients that were not treated by PT had a low burden of disease compared to the group of patients treated by a PT. Indeed, these patients had a double burden of disease (inactive and significantly more exacerbations) or even a triple burden of disease (inactive, significantly more exacerbations and more vascular comorbidity).

Thus, the hypotheses that the referral of patients to PT is based on the patients' burden of disease and that this is not necessarily coherent with the level of inactivity and that patient phenotypes are unequal in different primary care settings were confirmed by the study results.

### 4.1. Considerations in Patient Referral

Although based on patient reported data only, this study confirmed that the majority of patients with COPD are inactive. The large proportion of patients within PT practice who are inactive (69%) is not surprising, since GP practice guidelines advise referring the patient to a PT if the physical activity standard is not achieved [14]. Interestingly, however, this study showed also that the group of inactive patients that was not treated by a PT is extremely large (74%). This can be clarified by different reasons. A GP might not refer patients to PT when physical training is not a feasible option. Alternatively, GPs might consider other patient characteristics needed for referral to PT than inactivity alone.

Solely based on the inactivity referral criteria, it means that the GP could have referred more patients to a PT. However, this statement needs some consideration. According to the patient reported outcomes, 73% of patients with COPD registered by the GP were physically inactive, while not more than 20% were referred to and treated by a PT. On the one hand, the respondents are a relatively small subgroup (35%) of the GP population, from which they were recruited. It is possible that patients who were treated by a PT responded less often to the questionnaire compared to patients who were not treated by a PT. However, the National Primary Care Collaboration LESA reported that 27% of patients with COPD were referred to a PT in one year [15], which is a number approximating the patients reported percentage (20%).

On the other hand, GPs might not have referred* inactive* patients to a PT when they showed no unfavourable prognosis based on other criteria such as exacerbations, comorbidity, or limitations in activity. Reversely, by taking into account these other criteria, GPs might have referred* active* patients that showed an overall higher burden of disease. Our findings confirmed the latter hypothesis, since referred patients to PT had higher exacerbation rates, more vascular comorbidity, higher degree of airway obstruction, worse symptom scores (CCQ-subscale symptoms), more limitation in daily activity (CCQ-subscale functional state), and lower health perception (GPE) or health status (CCQ-total scale).

### 4.2. Clinical Implication of Phenotypic Variation

The phenotype of patient populations in different primary care settings varies. This finding may have several clinical implications.

The results of this study can provide both GPs and PTs with a realistic perspective from which prior expectations are set and treatment results are being evaluated. In the light of (potential) referral criteria, it is useful to understand why part of the inactive patient population is not referred to or treated by PT.

This study increases GPs awareness of the phenotypes of patients treated by a PT. Our data also shows that GPs might deviate from the GP guidelines regarding referral criteria for good reasons such as disease-related criteria mentioned in the GOLD report [1]. GPs consider comorbidity as an important part of COPD management, including referring patients to a PT. This is a relevant finding, since it is apparent that COPD clinicians should focus their attention not only on the management of COPD itself, but also on the investigation and management of COPD comorbidities [30].

For physical therapists it is important to have insight into the phenotype of COPD patients who receive PT in terms of tailoring their clinical reasoning and treatment. This study showed that PTs, treating patients with COPD, cope with a patient population that has a relatively higher burden of disease compared to the patient population treated by a GP only. This insight is also necessary to improve COPD care workflows in primary care in order to achieve proactive maintenance instead of acute rescue in COPD management [31].

Patients may not seek medical attention until their symptoms become troublesome and persistent and significant respiratory impairment and comorbidities are present [32]. The more severe and complex patient population in the PT practice may be one of the reasons that PTs treat patients with COPD for long-term periods. Studies with long-term exercise programmes for patients with COPD generally achieve more favourable results regarding functional exercise capacity, skeletal muscle function, and health-related quality of life [33]. Although long-term exercise programmes are more expensive and take more effort for patients, neither health care insurance companies nor patients are well served by programmes that yield only modest benefit [33].

Health care insurance companies should bear in mind the existence of phenotypic variations in their target population before comparing and judging treatment results across different primary care settings. Patients with COPD who are treated in PT practices are more complex and may need longer treatment because of their higher burden of disease (more exacerbations, more comorbidity, and lower quality of life). Moreover, health care insurers better not base the reimbursement for PT in COPD solely on the degree of airway obstruction (GOLD stage). Parameters that define the burden of disease and those that can be improved by PT (as part of pulmonary rehabilitation) should be taken into account to determine reimbursement policy. Exacerbation frequency [34, 35], limitations in daily activities [35–37], and comorbidities, but not necessarily airway obstruction [38], should be considered as criteria for PT reimbursement [39].

From the patients' perspective, it seems favourable to be treated by a PT earlier in the disease process, which can yield favourable results like higher functional exercise capacity (walk distance), more muscle strength, quality of live (mastery), and daily physical activity (steps) [40]. Referral to PT should not be delayed until their activity rate has dropped below threshold (international standard for physical activity) and their burden of disease is high enough (e.g., only patients with a forced expiratory volume in one second (FEV_1_) of 50% of the predicted or higher are eligible for PT reimbursement in Netherlands). It is important to assess and encourage physical activity in the earliest stages of COPD in order to maintain a physical activity level that is as high as possible, as this is associated with better prognosis [2].

### 4.3. Limitations of the Study

The response rate in this study was 35%. Compared to studies that used paper-based questionnaires that reached response rates within 33–75%  [41], our response rate can be considered relatively low. Response rates are probably more dependent on the population sampled than on any other factor [42]. The questionnaire was combined with multiple questions about smoking for the benefit of another study. The number of patients with COPD who did not want to fill out these specific questions may have reduced the response rate. Since we are unable to compare the patient characteristics of the nonrespondents with the respondents, it is important that our 35% is a representative sample of the base population [41]. The distribution of GOLD stages in this study is comparable with population-based samples mentioned in international literature [43]. The percentages of the comorbidities present in our study correspond relatively well with other COPD populations [1, 44]. The number of exacerbations is slightly higher than the populations mentioned by the Global Initiative for Chronic Obstructive Lung Disease, whereas the number of hospitalisations is similar per GOLD stage [1]. The respondent characteristics in this study approximate the characteristics of the COPD population described in the literature. Nevertheless, the statistical significance of the specific differences between the subpopulations in this study needs to be interpreted with care as the external validity can be compromised [45].

Due to the transversal study design, no causal effect can be assured for the influence of other referral criteria on the actual referral by GPs. However, the aim of this study was to reveal any differences in phenotypes between patients with COPD treated by PT versus GP only, and this was answered with the present study design. The demonstrated higher burden of disease can be seen as a reason for referral to PT. Indeed, it is less likely that a higher burden of disease emerges as a consequence of PT, since PT has shown its effect on reduced hospital admission and mortality and improved health-related quality of life in COPD in other studies [35]. The higher burden of disease in the group treated by GP combined with PT was accompanied by the remarkable lower smoking rate and higher physical activity rate. It is possible that the physical activity rate was increased after referral to PT and not vice versa. Some studies showed a significant increase in daily physical activity after pulmonary rehabilitation; however other studies did not find an increase in the level of physical activity [46]. Moreover, once or twice a week guided therapy for COPD (which includes at least 30 minutes of moderate exercise) will not necessarily increase the patient reported physical activity rate per week to cross the inactivity threshold of the physical activity standard.

Another limitation might be the use of questionnaires, introducing potentially social desirable answers. But patient reported outcome measures cannot be left out when determining differences between patients' burdens of disease in primary care. It has been shown that perceptions of limitations and reported limitations are a stronger predictor of behaviour or disease severity than objective measures of severity [16, 18].

## 5. Conclusion

General practitioners treat inactive patients with COPD who are not referred to or treated by a PT. Inactive patients treated by a GP combined with a PT differ significantly from those treated by a GP only. The COPD patient population in PT practices showed a higher burden of disease, regarding higher exacerbations rates, more vascular comorbidity, more severe airway obstruction, worse symptoms, more limitations in daily activity, and, consequently, lower health perception or health status. Besides the specified inactivity criterion in GP guidelines, these factors may play a role in the referral to physical therapy by a GP. These observations have implications for clinical expectations regarding therapy outcomes, for the way health care reimbursement for PT is organised and for generalizability of study results in future research.

## Figures and Tables

**Table 1:** Characteristics of 290 inactive patients with COPD treated in primary care.

Characteristic	Treated by GP only (*n* = 236)	Treated by GP and PT for COPD (*n* = 54)	*p* value
Sex, *n* male (%)	122 (52)	31 (57)	0.55
Age (yr), mean (SD)	70.29 (11.07)	70.87 (8.76)	0.72
Smoking (pack years), mean (SD)	31.14 (23.54)	40.90 (23.39)	0.001
GOLD stage, *n* (%)			<0.0001
I	92 (52)	5 (11)	
II	68 (39)	18 (39)	
III	10 (6)	15 (33)	
IV	6 (3)	8 (17)	
Comorbidity, *n* (%)			
Cardiovascular	71 (44)	21 (39)	0.317
Cardiac	51 (22)	13 (24)	0.543
Vascular	21 (9)	11 (20)	0.020
Stroke	11 (5)	5 (9)	0.160
Respiratory (asthma)	26 (11)	5 (9)	0.456
Psychological (depression)	32 (14)	5 (9)	0.226
Metabolic (diabetes)	35 (15)	12 (22)	0.137
Nutritional	64 (27)	15 (28)	0.536
Exacerbations in the past year, *n* (%)			0.001
0	127 (54)	16 (30)	
1; of which hospitalised	55 (23); 7 (13)	12 (22); 4 (33)	
2; of which hospitalised	24 (10); 1 (4)	9 (17); 1 (11)	
3 or more; of which hospitalised	29 (12); 15 (52)	17 (32); 8 (47)	
Disease-related health status (0–6), mean (SD)			
Total CCQ	1.40 (0.95)	1.80 (0.95)	<0.0001
Symptoms subscale	1.85 (1.11)	2.35 (1.07)	0.004
Functional state subscale	1.32 (1.14)	2.40 (1.25)	<0.0001
Mental state subscale	0.68 (0.99)	0.92 (1.02)	0.038
General health perception (0–5), *n* (%)			<0.0001
1, excellent	1 (1)	1 (2)	
2, very good	8 (3)	0	
3, good	96 (45)	9 (17)	
4, moderate	90 (43)	35 (66)	
5, poor	17 (8)	8 (15)	

GP: general practitioner; PT: physical therapist; SD: standard deviation; GOLD: the Global Initiative for Chronic Obstructive Lung Disease; GOLD stages: I: mild COPD, FEV_1_/FVC < 0.7, and FEV_1 _≥ 80% of predicted; II: moderate COPD, FEV_1_/FVC < 0.7, and 50% ≤ FEV_1_ < 80% of predicted; III: severe COPD, FEV_1_/FVC < 0.7, and 30% ≤ FEV_1_ < 50% of predicted; IV: very severe COPD, FEV_1_/FVC < 0.7, and FEV_1_ < 30% of predicted or FEV_1_ < 50% of predicted plus chronic respiratory failure; FVC: postbronchodilator forced vital capacity; FEV_1_: postbronchodilator forced expiratory volume in one second; CCQ: Clinical COPD Questionnaire: rating from 0 “good” to 6 “bad.”

**Table 2:** Characteristics of all 438 patients with COPD treated in primary care.

Characteristic	All patients (*n* = 438)	Treated by GP only (*n* = 318)	Treated by GP and PT for COPD (*n* = 78)	*p* value
Sex, *n* male (%)	235 (54)	166 (52)	45 (58)	0.71
Age (yr), mean (SD)	69 (11)	69 (11)	69 (11)	0.29
Physical activity (0–8), mean (SD)	2.38 (2.31)	2.20 (2.33)	2.92 (1.99)	0.001
Smoking (pack years), mean (SD)	31.14 (23.54)	30.45 (23.30)	38.27 (22.32)	0.004
GOLD stage, *n* (%)				<0.0001
I	141 (32)	133 (55)	5 (7)	
II	116 (27)	88 (36)	27 (41)	
III	35 (8)	14 (6)	21 (32)	
IV	20 (5)	7 (3)	13 (20)	
Comorbidity, *n* (%)				
Cardiovascular	116 (27)	88 (26)	25 (32)	0.171
Cardiac	82 (19)	61 (19)	16 (21)	0.440
Vascular	38 (9)	22 (7)	13 (17)	0.009
Stroke	20 (5)	14 (4)	6 (8)	0.177
Respiratory (asthma)	56 (13)	44 (14)	9 (12)	0.381
Psychological (depression)	60 (14)	44 (14)	10 (13)	0.499
Metabolic (diabetes)	70 (16)	50 (16)	13 (17)	0.469
Nutritional	115 (27)	87 (28)	23 (30)	0.390
Exacerbations in the past year, *n* (%)				<0.0001
0	204 (51)	181 (57)	22 (28)	
1; of which hospitalised	89 (22); 12 (13)	70 (22); 8 (11)	18 (23); 4 (22)	
2; of which hospitalised	47 (12); 4 (9)	32 (10); 3 (9)	14 (18); 1 (8)	
3; of which hospitalised	19 (5); 6 (32)	14 (5); 5 (36)	5 (7); 1 (20)	
more than 3; of which hospitalised	39 (10); 21 (54)	19 (6); 11 (58)	19 (24); 10 (53)	
Disease-related health status (0–6), mean (SD)				
Total CCQ	1.51 (1.06)	1.32 (0.97)	2.19 (1.01)	<0.0001
Symptoms subscale	1.95 (1.19)	1.76 (1.14)	2.49 (1.13)	<0.0001
Functional state subscale	1.52 (1.28)	1.20 (1.13)	2.48 (1.29)	<0.0001
Mental state subscale	0.75 (1.06)	0.67 (1.00)	1.02 (1.13)	0.004
General health perception (0–5), *n* (%)				<0.0001
1, excellent	6 (2)	4 (1)	1 (1)	
2, very good	13 (3)	11 (4)	0	
3, good	168 (42)	133 (47)	15 (20)	
4, moderate	172 (44)	112 (39)	51 (66)	
5, poor	37 (9)	26 (9)	10 (13)	

GP: general practitioner; PT: physical therapist; SD: standard deviation; physical activity: rating from 0 “not physically active” to 8 “very physically active”; GOLD: the Global Initiative for Chronic Obstructive Lung Disease; GOLD stages: I: mild COPD, FEV_1_/FVC < 0.7, and FEV_1_ ≥ 80% of predicted; II: moderate COPD, FEV_1_/FVC < 0.7, and 50% ≤ FEV_1_ < 80% of predicted; III: severe COPD, FEV_1_/FVC < 0.7, and 30% ≤ FEV_1_ < 50% of predicted; IV: very severe COPD, FEV_1_/FVC < 0.7, and FEV_1_ < 30% of predicted or FEV_1_ < 50% of predicted plus chronic respiratory failure; FVC: postbronchodilator forced vital capacity; FEV_1_: postbronchodilator forced expiratory volume in one second; CCQ: Clinical COPD Questionnaire: rating from 0 “good” to 6 “bad.”
